# Residue, Dissipation Pattern, and Dietary Risk Assessment of Imidacloprid in Chinese Chives

**DOI:** 10.3389/fnut.2022.846333

**Published:** 2022-02-23

**Authors:** Rongqi Zhai, Kaige Zhang, Ge Chen, Guangyang Liu, Xiaodong Huang, Mingkun Gao, Jie Zhou, Xiaomin Xu, Lingyun Li, Yanguo Zhang, Jing Wang, Maojun Jin, Donghui Xu, A. M. Abd El-Aty

**Affiliations:** ^1^Key Laboratory of Vegetables Quality and Safety Control, Laboratory of Quality & Safety Risk Assessment for Vegetable Products, Institute of Vegetables and Flowers, Ministry of Agriculture and Rural Affairs, Chinese Academy of Agricultural Sciences, Beijing, China; ^2^Key Laboratory of Agro-Product Quality and Safety, Institute of Quality Standard and Testing Technology for Agro-Products, Ministry of Agriculture and Rural Affairs, Chinese Academy of Agricultural Sciences, Beijing, China; ^3^Department of Pharmacology, Faculty of Veterinary Medicine, Cairo University, Giza, Egypt; ^4^Department of Medical Pharmacology, Faculty of Medicine, Atatürk University, Erzurum, Turkey

**Keywords:** imidacloprid pesticides, dissipation dynamics, Chinese chives, sample preparation, risk assessment

## Abstract

The demand for Chinese chives is growing as they are also rich in vitamins, fiber, and sulfur nutrients. Chinese chives should be sprayed with imidacloprid to control pests and diseases to safeguard their yield and to meet the demands of East Asian consumers for Chinese chives. Overspraying of imidacloprid can lead to residues in Chinese chives, posing a severe risk to human health. To reduce the harmful effects of imidacloprid residues on humans, we investigated the imidacloprid dissipation pattern and the final residue on Chinese chives using the quick, easy, cheap, effective, rugged, and safe (QuEChERS) method combined with liquid chromatography-tandem mass spectrometry (LC-MS/MS). Good linearity (*R*^2^= 0.9988), accuracy (expressed as recovery % of 78.34–91.17%), precision [expressed as relative SDs (RSDs) of 0.48–6.43%], and sensitivity [a limit of quantification (LOQ) ≤ 8.07 × 10^4^ mg/kg] were achieved. The dissipation dynamics were consistent with the first-order kinetics, with a half-life of 2.92 days. The final residual levels on Chinese chives were 0.00923–0.166 mg/kg, which is lower than the maximum residue limits (MRLs) of 1 mg/kg for imidacloprid on Chinese chives. A risk assessment index of <1 indicates that Chinese chives are safe for consumption.

## Introduction

Chinese chives (*Allium tuberosum*) are among the most important vegetables to East Asians (1). It is rich in vitamins, fiber, and sulfur compounds with antiseptic properties (2, 3). According to traditional Chinese medicine, it has aphrodisiac, anti-cancer, antioxidant, and other healing properties and treats abdominal pain and asthma (4–6). Modern medicine shows that the dietary fiber in Chinese chives could promote intestinal peristalsis and accelerate the body's metabolism, thereby preventing colorectal cancer. It also decreases cholesterol absorption and prevents atherosclerosis and coronary heart disease (7, 8). Therefore, the economic value of chives is increasing, which leads to the expansion of the planting area.

As Chinese chives are planted on a large scale, plant pests and diseases increased accordingly. The Chinese chives maggot (*Bradysiaodoriphaga*), with a short reproductive cycle, high fertility, and overwintering under protected conditions, becomes an annual pest occurrence (9). The rate of affected plants can reach 20–50%. The damage of Chinese chives maggot is the most severe problem (10). Farmers spray imidacloprid to control pests, which acts as an inhibitor of nicotinic acetylcholine receptors (nAChRs) in the central nervous system of insects, causing disruption of the insect's nervous system and, eventually, leading to death (11, 12). It fails to decompose thoroughly, resulting in long residual levels (13) that would result in harmful health effects.

The residue dissipation of imidacloprid has been reported in Chinese chives. However, few studies detected the imidacloprid residues using a liquid chromatography-tandem mass spectrometry (LC-MS/MS), and assessed its dietary risk in Chinese chives (14, 15). Studying pesticide residues and dissipation of imidacloprid is vital in Chinese chives because of its high residual pesticide and less-relevant dissipation dynamics (16). This study developed a method to analyze the imidacloprid residues in Chinese chives using QuEChERS combined with LC-MS/MS. Furthermore, we determined the dissipation pattern and the dietary risk assessment to help planters to further master the spraying and the harvesting period of imidacloprid in Chinese chives, thus, reducing the risk to the environment and the human health. Secondly, the study further explored the quick sample preparation of imidacloprid in Chinese chives. Finally, the study compared the recoveries, relative SDs (RSDs) of QuEChERS, and quick extraction to select a more suitable sample preparation for imidacloprid in Chinese chives.

## Materials and Methods

### Chemicals and Equipment

Imidacloprid, water-dispersible granules (10% WG), was obtained from Hebei Noda Agrochemical Co., Ltd. Imidacloprid standard (100 mg/kg), was procured from Beijing Manhage Bio-Technology Co. Ltd. Acetonitrile (chromatographic grade), and was purchased from Merck AG, Germany. Anhydrous magnesium sulfate (analytical grade) was secured from Xilong Chemical Co., Ltd. Sodium chloride (analytical grade) and was supplied by Tianjin Huihang Chemical Technology Co., Ltd. Dispersed solid-phase extraction purifier (52-mg PSA; 52-mg C18; 26-mg GCB) was obtained from Shimadzu Corp. (Kyoto, Japan).

### Field Experiment and Sampling

Final residue and dissipation field trials were carried out following the “Guideline for Testing of Pesticide Residues in Crops” (NY/T 788–2018) (Ministry of Agriculture and Rural Affairs of the People's Republic of China, 2018). The final residual experiments were conducted in May 2020 at Tongzhou Beijing. The experiment set up a protective belt around each 50-m^2^ plot, including a control plot without application of imidacloprid, an experiment plot of imidacloprid dissipation, and an experiment plot of final imidacloprid residue was reserved during the whole growth period.

Imidacloprid, at 10% WG, was applied at a dosage of 0.429 g/L when Chinese chives grew to 10–30 cm to investigate its dissipation. Fresh samples of at least 2 kg Chinese chives that have been drugged and are growing normally were randomly collected from 24 points of each plot. The fresh samples were collected and packed into sample containers that were wrapped and labeled after 2 h, 8 days, 18 days, and 25 days following the last application.

Soil and wilted leaves attached to the Chinese chives were removed. The samples were cut and mixed, homogenized with a beater, and packed into sample boxes. The sample boxes were labeled and stored at −20°C in the refrigerator.

### Sample Preparation

As the composition of Chinese chives substrate is more complex than other vegetables, the sample preparation was carried out with two different methods.

Method 1 (QuEChERS): Chinese chives homogenate (10 g) was weighed and placed in a 50-mL centrifuge tube. Then, 10-mL acetonitrile was added to the centrifuge tube and vortexed for 1 min. After that, 4-g anhydrous magnesium sulfate and 1-g sodium chloride were added, vortexed again for 1 min, and centrifuged at 6,000 r/min for 5 min. The supernatant (9 mL) was transferred to a 50 mL centrifuge tube containing 90-mg dispersed solid-phase extraction purifier, vortexed for 30 s, and centrifuged at 6,000 r/min for 2 min. The sample was filtered through a 0.22-μm membrane and transferred into a glass vial for LC-MS/MS.

Method 2 (quick extraction): Chinese chives homogenate (1 g) was weighed and was placed in a 10-mL centrifuge tube. Next, 4 mL of an aqueous solution of 2, 5, and 10% of methanol were added respectively to the different centrifuge tubes, shaken manually for 30 s, and left undisturbed for 1 min. The sample was filtered through a 0.22-μm membrane and transferred into a glass vial for LC-MS/MS.

### Liquid Chromatography-Tandem Mass Spectrometry (LC-MS/MS)

A Shimadzu LC-30A, MS8050 HPLC–MS/MS (Shimadzu Corp., Kyoto, Japan) instrument, equipped with an electrospray ionization (ESI), was adopted here. A Phenomenex-C18 column (50 mm × 3 mm, 2.6 μm) with an injection volume of 1 μL; column temperature of 40°C; flow rate of 0.3 ml/min; and elution conditions are shown in Table 1. The mass spectrometer was operated with electrospray in the positive ion mode (ESI^+^), and the ions were monitored in the multiple reaction monitoring (MRM) mode. Mass spectrometric conditions: ion source temperature 400°C; ion pairs and collision energy and other parameters are shown in Table 2.

**Table 1:** Imidacloprid elution conditions.

**Time**	**Flow rate**	**Methanol**	**Ammonium acetate**
**(min)**	**(mL/min)**	**(%)**	**solution (1 mmol,%)**
0	0.3	20	80
8	0.3	95	5
12	0.3	95	5
12.1	0.3	20	80

**Table 2:** Characteristic monitoring ion of imidacloprid.

**Name**	**Qualitative ion (*m/z*)**	**Quantification ion(*m/z*)**	**Retention time /min**	**Q1 Pre bias/V**	**Q3 Pre bias /V**	**Collision energy /eV**
Imidacloprid	256.10/175.10	256.10/209.10	2.701	−27 −27	−27 −22	−16 −14

### Dissipation Kinetics

The residue levels of imidacloprid on Chinese chives decreased with application time in Chinese chives, expressed by the first-order kinetic equation:


(1) $$\begin{array}{r} C_{t}=C_{0}e^{-kt} \end{array}$$

(2) $$\begin{array}{r} T_{1/2}=\ln2/k \end{array}$$

where C_t_ and C_0_ represent the pesticide residual levels (mg/kg) at times t and 0 (days), respectively.

### Dietary Exposure Assessment

We assessed the risk of imidacloprid in Chinese chives using a chronic dietary risk quotient (RQc) based on a risk factor approach (17).


(3) $$\begin{array}{r} RQc=\frac{NEDI}{ADI} \end{array}$$

(4) $$\begin{array}{r} NEDI=\frac{\sum \left(Fi\times STMR\right)}{bw} \end{array}$$

NEDI (mg/kg· bw/day): the national estimated daily intake; Fi (kg/day): the average daily *per capita* consumption of a particular food in China; STMR (mg/kg): supervised trials median residue; bw: the average body weight of Chinese adults (63 kg); ADI (mg/kg bw/day): the acceptable daily intake.


(5) $$\begin{array}{r} RQa=\frac{NESTI}{ARfD} \end{array}$$

(6) $$\begin{array}{r} NESTI=\frac{LP\times HR}{bw} \end{array}$$

NESTI (mg/kg·bw/day): the national estimated short-term intake; LP (kg/day): the large portion of specific food item consumed per day; HR (mg/kg): the highest residue; ARfD (mg/kg·bw/day): the acute reference dose. ARfD of imidacloprid was established to be 0.4 mg/kg·bw/day.

According to Mao et al. (18), the *per capita* annual consumption of Chinese chives was 33.40 kg, and the *Fi* of Chinese chives was 0.0915 kg/day. As the LP of Chinese chives was not reported, it has to be replaced by dark green vegetables (LP = 0.5 kg) (19). The RQ <1 and RQ > 1 indicate whether the risk of the evaluated pesticide is acceptable and unacceptable to consumers, respectively.

## Results and Discussion

### Method Validation

**Figure 1:**
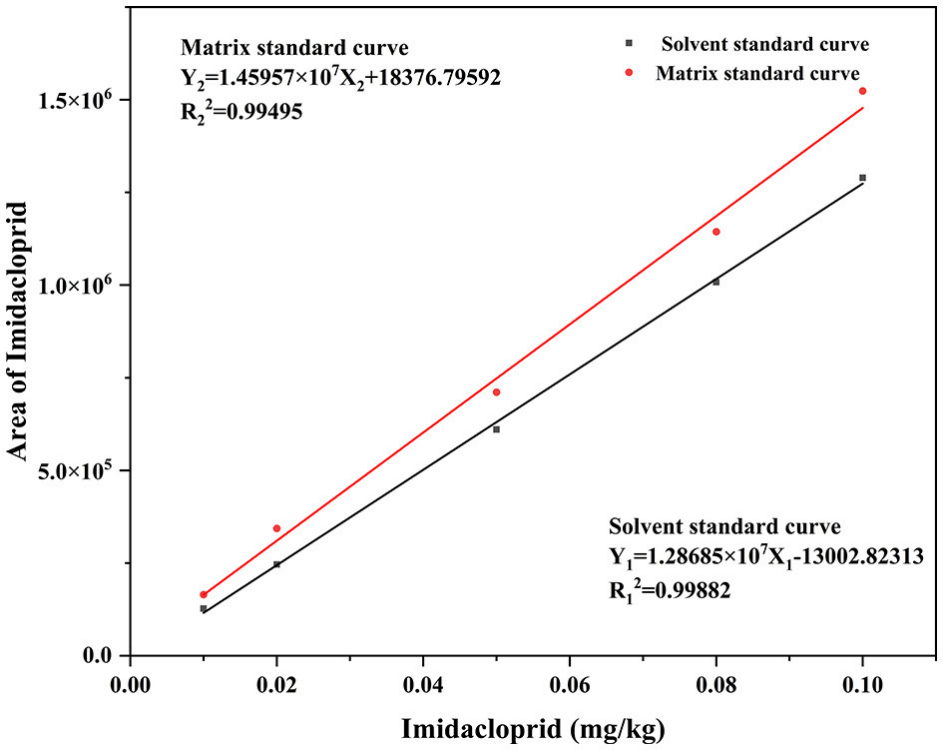
Standard curve of imidacloprid in Chinese chive.

According to NY/T 788-2018, the analytical method was validated using accuracy, precision, quantification limit, detection limit, matrix effect (ME), and linearity. The method's linearity was evaluated by plotting matrix-matched calibration curves at five concentrations within 0.01–0.10 mg/kg. The ME represents the ratio of the slope of the matrix matching curve to the slope of the solvent curve. The values >1 and <1 represent a signal enhancement and suppression effects, respectively (20). The imidacloprid standard was prepared in acetonitrile and blank solution at a concentration rate of 0.01, 0.02, 0.05, 0.08, and 0.1 mg/kg. The solvent and matrix-matched calibrations for imidacloprid are Y_1_ = 1.28685 × 10^7^X_1_ −13002.82313, *R*${}_{1}^{2}$= 0.99882 and Y_2_ = 1.45957 × 10^7^X_2_ + 18376.79592, *R*${}_{2}^{2}$= 0.99495 (Figure 1). The calculated ME was >1, denoting that the Chinese chives mechanism has an enhancement effect.

Accuracy and precision are expressed as recovery (recovery of 70–120%), and relative standard deviation (RSD ≤ 20%) is according to NY/T 788-2018 (21). The average recoveries of imidacloprid were 78.34–91.17%, with RSD between 0.48 and 6.43% using QuEChERS; the finding that met the NY/T788-2018 requirements. These results showed a satisfactory accuracy and precision of imidacloprid in Chinese chives matrices.

### Comparison of QuEChERS and Quick Extraction

**Figure 2:**
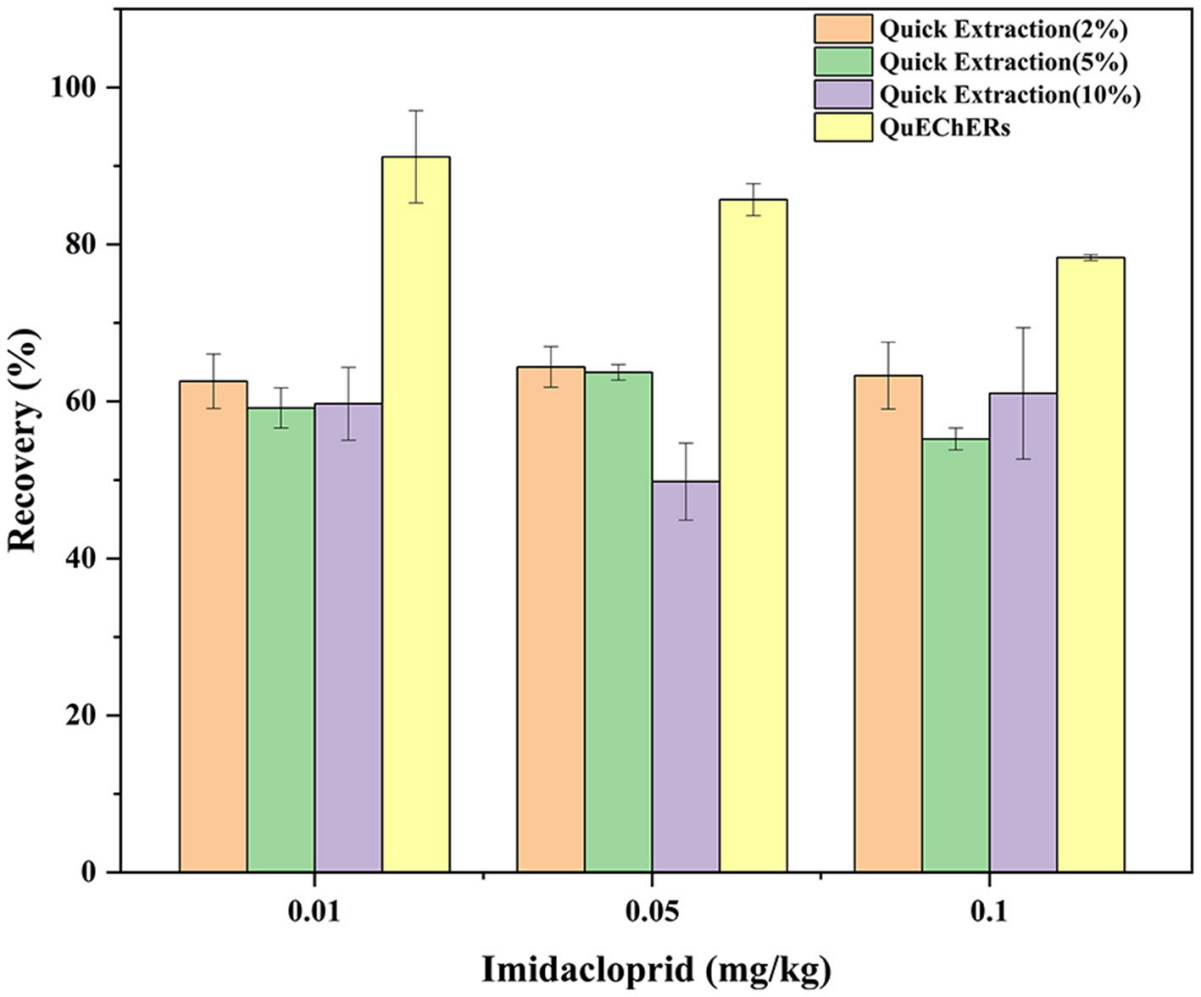
Average recovery of spiked levels using QuEChERS and Quick extraction in Chinese chive.

**Figure 3:**
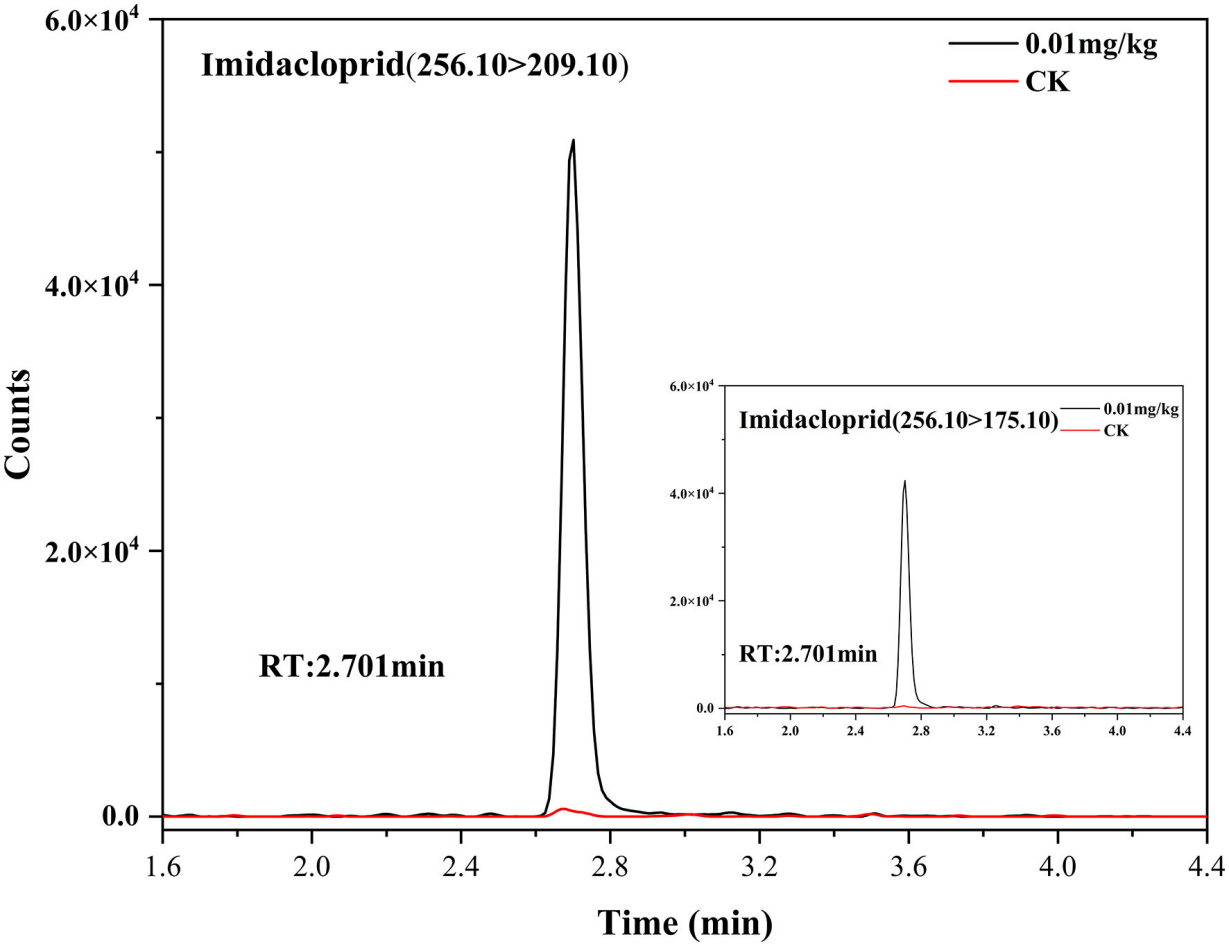
Extracted ion chromatograms of imidacloprid in control and spiked sample (0.01 mg/kg) for Chinese chive.

The extraction efficiency (expressed as recovery ± RSD) of the 2 tested methods was assessed by spiking three different concentration levels (0.01, 0.05, and 0.1 mg/kg) of imidacloprid to a blank matrix in six replicates (*n* = 6)(Figure 2). The results are summarized in Table 3. We tested acetonitrile, ethyl acetate, acetone, and methanol as an extraction solvent to determine the imidacloprid residues in agricultural products (22). It has to be noted that acetonitrile is more expensive and toxic, ethyl acetate is not miscible with an aqueous solution, and acetone requires availability to purchase the organic solvents. Hence, all the above organic solvents were excluded (23–25). We have chosen methanol as a quick extraction solvent. Methanol has low environmental toxicity, cheap, and easy to obtain. To obtain a satisfactory recovery, different ratios of aqueous methanol solutions were optimized. The average recoveries of imidacloprid were 62.57–64.40%, 55.22–63.71%, and 49.80–61.03% with RSD between 4.02 and 6.73%, 1.55 and 4.31%, and 7.78 and 13.71% using an aqueous solution of 2, 5, and 10% methanol, respectively. The recovery is inconsistent with the standard NY/T 788-2018 using methanol aqueous solution as an extractant. Thus, the ratio and composition of the extractant need to be further improved. On the other hand, the average recoveries of imidacloprid were 78.34–91.17%, with RSD between 0.48 and 6.43% using QuEChERS in Chinese chives, which met the experimental criteria. The extracted ion chromatograms of imidacloprid in blank and spiked samples are shown in Figure 3. The following method validation and dissipation dynamics were performed using QuEChERS.

**Table 3:** Recoveries and relative standard deviations (RSD) of imidacloprid in Chinese chives.

**Spiked**	**QuEChERS**		**Quick extraction**					
**(mg/kg)**			**Methanol (2%)**		**Methanol (5%)**		**Methanol (10%)**	
	**Recovery (%)**	**RSD (%)**	**Recovery (%)**	**RSD (%)**	**Recovery (%)**	**RSD (%)**	**Recovery (%)**	**RSD (%)**
0.01	91.17	6.43	62.57	5.54	59.18	4.31	59.71	7.78
0.05	85.72	2.37	64.40	4.02	63.71	1.55	49.80	9.85
0.1	78.34	0.48	63.29	6.73	55.22	2.53	61.03	13.71

In QuEChERS, the adsorbents PSA, C-18, and GCB were used to adsorb organic acids, vitamins, and pigments. Moreover, anhydrous magnesium sulfate and sodium chloride were used to remove the water from the samples (22, 26). Most importantly, extraction with 100% acetonitrile results in higher recoveries. The quick extraction method used different ratios of methanolic aqueous solutions without purification. The steps of salting out and purification had less effect on the extraction rate of imidacloprid in the QuEChERS method. The main reason for the high extraction rate of QuEChERS was that the acetonitrile is more polar than methanol, and its proportion was 100%. The proportion of organic solvent was higher, contributing to the high extraction efficiency of the QuEChERS method.

### Dissipation of Imidacloprid in Chinese Chives

**Figure 4:**
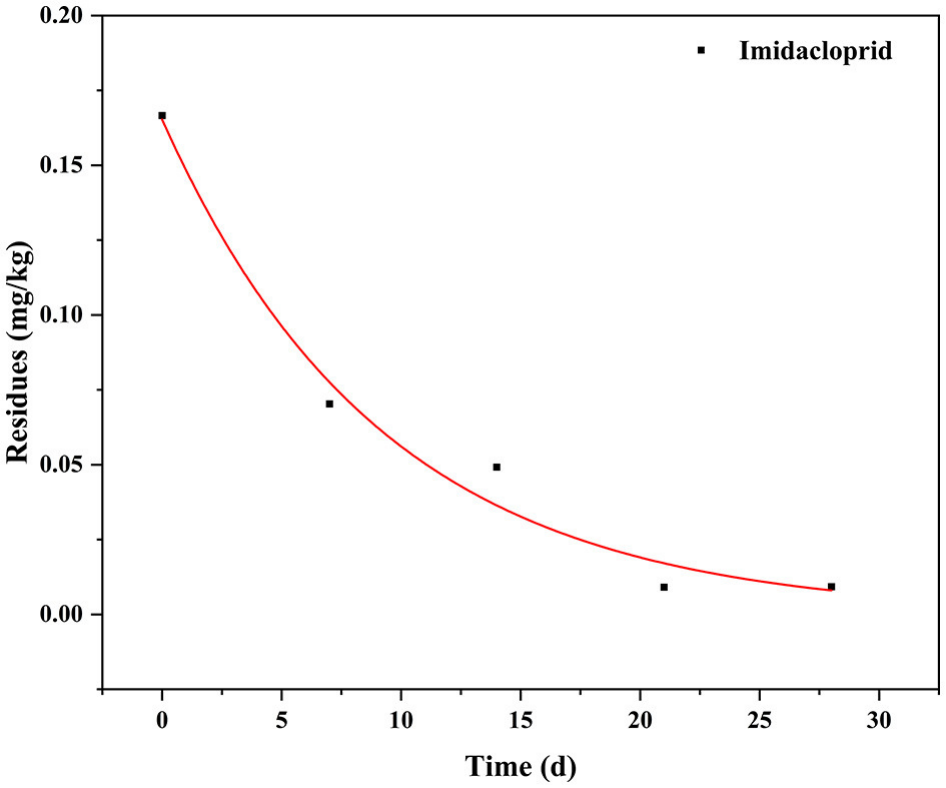
Dissipation curves of imidacloprid in Chinese chive.

The dissipation data and curves of imidacloprid in Chinese chives are shown in Table 4 and Figure 4. The dissipation dynamic under field conditions followed a first-order kinetics model, with correlation coefficients (*R*^2^) of 0.9831. The dissipation dynamics show a fast pre-late stage and a flat mid-stage. The initial residues were 0.1666 mg/kg with half-lives (T_1/2_) of 2.92 days. Approximately 90% of imidacloprid residues were dissipated 28 days after application. The dissipation dynamics suggested that imidacloprid is readily degraded in Chinese chives during its growth. Other experimental field studies have shown that the dissipation dynamics of imidacloprid are affected by various factors, including crop type, climate and environmental conditions, application dose and timing, and co-application with other pesticides (27, 28). In this context, Li et al. (29) investigated the final residues and the dissipation dynamics of imidacloprid in tomatoes by LC-MS/MS, with a half-life of 14.43 days and *R*^2^ = 0.90. Imidacloprid in tomatoes decreased rapidly during the first 7 days. It tended to decrease slowly after 14 days, consistent with a rapid degradation trend in the first period and a slight decrease in the second period. The same trend of imidacloprid dissipation was observed in other crops, as detailed in Table 5. The present study is consistent with previous studies, presenting trends in line with each other. Different crop types and environmental factors can influence the dissipation of imidacloprid. However, volatilization is the main factor of imidacloprid dissipation under outdoor conditions, leading to imidacloprid dissipation (37).

**Table 4:** Dissipation kinetic equations, half-lives, and other related parameters of imidacloprid in Chinese chives.

**Initial deposit**	**Dissipation kinetic**	**Correlation coefficient**	**Half-life**
**(mg/kg)**	**equations**	**(*R^**2**^*)**	**(d)**
0.1666	$C_{t}\;=\;165.290e^{-0.108t}$	*R^2^*= 0.9831	2.92

**Table 5:** Effect of different factors on imidacloprid dissipation.

**Crop type**	**Volume**	**Application times**	**Application interval (d)**	**Mix with other pesticides**	***R^**2**^***	**Half-time (d)**	**References**
Tomato	2.5 L ha^−1^	1	-	No	0.90	14.43	(29)
Rice	20 g ai ha^−1^	2	10	No	0.992	7.16	(30)
Brinjal	42 g ai ha^−1^	3	7	No	-	2.31	(31)
Cardamom	20 g ai ha^−1^	1	-	No	0.98	3.63	(32)
Green chilies	20 g ai ha^−1^	2	7	No	0.996	2.08	(33)
Tea	30 g ai ha^−1^	1	-	No	0.972	2.45	(34)
Cabbage	32.38 g ha^−1^	1	-	No	0.934	33.0	(35)
Celery	56.2 g ai ha^−1^	1	-	Yes	0.99	46.5	(36)

### Dietary Exposure Assessment

The acute and chronic dietary risk of total residues of imidacloprid in Chinese chives was assessed (Table 6). For a Chinese adult weighing 63 kg, the average consumption of Chinese chives was 0.0915 kg/d, and the ADI of imidacloprid was 0.06 mg/kg bw/day. According to Eqs. (3)–(4), the RQc of imidacloprid was 0.119–0.170% in Chinese chives, which is <100%. The large portion that is reported for Chinese chives was 0.5 kg/d. The highest residue of Chinese chives was 0.167 mg/kg. The ARfD of imidacloprid was established to be 0.4 mg/kg bw/day. According to Eqs. (5)-(6), the RQa of imidacloprid was 0.139–0.331% in Chinese chives, which is <100%. These results suggest that the acute and chronic dietary risk of imidacloprid is not threatening the health of the average Chinese consumers for Chinese chives.

**Table 6:** Dietary exposure risk assessment.

**Matrix**	**PHI**	**Residue**	**STMR**	**NEDI**	**RQc**	**HR**	**NESTI**	**RQa**
	**(d)**	**(mg/ kg)**	**(mg/ kg)**	**(mg/kg·bw/d)**	**(%)**	**(mg/kg)**	**(mg/kg bw/d)**	**(%)**
Chinese chives	7	0.0703	0.0703	0.000102	0.170	0.167	0.00132	0.331
	14	0.0492	0.0492	0.0000715	0.119	0.0703	0.000558	0.139

Moreover, the *Fi* ratio of different age groups to the body weight was significantly different. Therefore, 6 distinct groups were selected, representing young children (2–4 years old), young adults (18–30 years old), and elderly (60–70 years old) for risk assessment. The NEDI and RQc were calculated by combining data on diet, bodyweight of Chinese, and the final residues of imidacloprid in Chinese chives determined in this study (38). In addition, the highest STMR (0.0703 mg/kg) at the recommended PHI (7 days) in the final residues was selected for evaluation. Following the principle of risk maximization, the Fi of Chinese chives was calculated using the vegetable. Table 7 shows that the RQc was <100% and decreased gradually with age. The RQc of children (2–4 years old) was the highest. The dietary exposure of females was slightly higher than that of males in the same age group due to differences in body weight and dietary habits between the sexes.

**Table 7:** The exposure risk of imidacloprid among different age groups in China.

**Age**	**Gender ^**a**^**	**Fi(kg/d)**	**Bw(kg)**	**NEDI(mg/kg·bw/d)**	**RQc (%)**
2–4	M	0.2234	14.1	0.00111	1.86
	F	0.2234	13.4	0.00117	1.95
18–30	M	0.3550	60.5	0.000412	0.687
	F	0.3550	52.6	0.000474	0.791
60–70	M	0.3664	61.3	0.000420	0.700
	F	0.3664	54.3	0.000474	0.791

a *M, male; F, female*.

## Conclusion

Herein, imidacloprid residual levels and dissipation dynamics on Chinese chives under outdoor conditions were determined using QuERChERS preparation and LC-MS/MS based on the application dose, frequency, and harvest interval criteria implemented in NY/788-2018. Furthermore, risk assessment was performed according to the risk quotient method. The pattern of imidacloprid dissipation in Chinese chives was best fitted to first-order kinetics with a half-life of 2.92 days. A recommended dosage of 10% WG under outdoor conditions and a spray interval of 7 days will not cause any harm to humans.

## Data Availability Statement

The original contributions presented in the study are included in the article/supplementary material, further inquiries can be directed to the corresponding authors.

## Author Contributions

RZ, KZ, and GC: investigation and writing – original draft. GL, XH, MG, and JZ: investigation and visualization. XX, LL, and YZ: visualization and supervision. JW, MJ, DX, and AA: validation and writing – review and editing. All authors contributed to the article and approved the submitted version.

## Funding

This study was financially supported by Central Public-interest Scientific Institution Basal Research Fund, Chinese Academy of Agricultural Sciences (IVF-BRF2021020), the Agricultural Science and Technology Innovation Program of CAAS (CAAS-ZDRW202011, CAAS-TCX2019025-5), the China Agriculture Research System of MOF and MARA (CARS-23-E03), and the National Key Research Development Program of China (2020YFD1000300).

## Conflict of Interest

The authors declare that the research was conducted in the absence of any commercial or financial relationships that could be construed as a potential conflict of interest.

## Publisher's Note

All claims expressed in this article are solely those of the authors and do not necessarily represent those of their affiliated organizations, or those of the publisher, the editors and the reviewers. Any product that may be evaluated in this article, or claim that may be made by its manufacturer, is not guaranteed or endorsed by the publisher.
